# Complementing community science with xenomonitoring: Understanding the eco-epidemiology of *Dirofilaria immitis* infection in dogs and mosquitoes

**DOI:** 10.1186/s13071-025-06882-0

**Published:** 2025-06-20

**Authors:** Tamara Szentivanyi, Laura V. González, Ágnes Klein, Zoltán Soltész, László Z. Garamszegi

**Affiliations:** 1 HUN-REN Centre for Ecological Research, Alkotmány Road 4, 2163 Vácrátót, Hungary; 2 Université Paris Cité, 5 Rue Thomas Mann, 75013 Paris, France; 3 National Laboratory for Health Security, HUN-REN Centre for Ecological Research, Karolina Road 29, 1113 Budapest, Hungary

**Keywords:** Citizen science, Dirofilariosis, Domestic dogs, Infection, Mosquito

## Abstract

**Background:**

Dirofilariosis is an emerging mosquito-borne disease that particularly affects domestic dogs worldwide but also causes symptoms in humans. Monitoring the distribution of emerging pathogens is essential for understanding the environmental and ecological factors influencing their transmission, which can be used to develop better prevention strategies.

**Methods:**

We applied both community science and molecular xenomonitoring to assess the occurrence of *Dirofilaria immitis* in domestic dogs and mosquitoes.

**Results:**

As part of the community science approach, we collected infection data from 1491 dogs from owners across Hungary, using a questionnaire survey. We found that 321 dogs (21.5%) tested positive for current or past dirofilariosis infection, with the highest prevalence observed in the southeastern (47.8%) and the eastern regions (43.4%) of the country. Age and living conditions affected infection status, with older dogs (aged 5–10 years and over 10 years) and those kept exclusively outdoors showing significantly higher infection rates. Molecular xenomonitoring revealed *D. immitis* infection in *Aedes albopictus*, *Aedes koreicus*, and *Aedes vexans*, with the highest minimum infection rate (MIR) in *Ae. koreicus* (28.5). Similar to community science results, the highest infection rates were observed in the southeastern and eastern regions (MIR: 14.9 and 11.6, respectively), but the two approaches generally provided overall similar geographical patterns.

**Conclusions:**

While xenomonitoring did not detect infections in Central Transdanubia, community science successfully provided host infection data, demonstrating its usefulness in assessing the presence and distribution of the disease. Finally, we emphasize the value of using an integrative approach, combining community science and xenomonitoring for monitoring dirofilariosis, especially in areas where direct pathogen screening is unavailable.

**Graphical Abstract:**

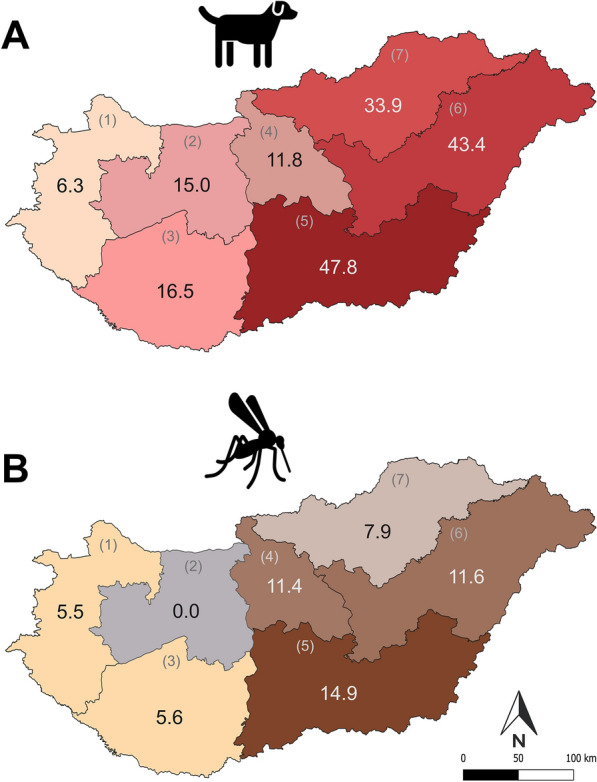

**Supplementary Information:**

The online version contains supplementary material available at 10.1186/s13071-025-06882-0.

## Background

Dirofilariosis is a parasitic disease in domestic dogs, cats, and, occasionally, in other mammals, including wild carnivores and humans [1, 2]. This vector-borne infection is transmitted by mosquitoes, causing mild to severe disease depending on the parasite species [3]. The two most frequently found species are *Dirofilaria immitis*, which is associated with heartworm disease and occurs worldwide, and *Dirofilaria repens*, which is the causative agent of subcutaneous dirofilariosis distributed in the Eastern Hemisphere [4]. The occurrence of dirofilariosis, driven by a complex interplay of ecological, climatic, and socioeconomic factors, poses significant challenges to both veterinarians and public health officials [4–6]. Effective control and management of this disease relies on continuous data collection and monitoring, which traditionally involve veterinary clinics and research institutions.

The emergence of dirofilariosis in domestic dogs is evident in several European regions, including Hungary [5, 7–10]. While the first occurrence of an autochthonous case of *D. immitis* in Hungary was reported in 2007 [11], the infection rate has shown a stable increase in the dog population since then [5, 11, 12]. The prevalence of dirofilariosis may vary depending on the diagnostic methods used and can differ across geographical regions [12, 13]. However, the average prevalence was 2.7% between 2011 and 2015 [11], but it increased to 11.3% by 2017 [5]. Furthermore, the infection rate in mosquitoes has also been reported to be increasing over the past years across Europe [3, 14–16].

Besides veterinarian testing, the distribution and occurrence of *Dirofilaria* species can also be monitored by molecular xenomonitoring of mosquitoes [17]. *Dirofilaria immitis* and *D. repens* have been detected in several mosquito species in Hungary and neighboring countries, including *Aedes vexans*, *Aedes cinereus*, *Anopheles hyrcanus*, *Anopheles maculipennis*, *Coquillettidia richiardii*, *Culex modestus*, *Culex pipiens*, *Ochlerotatus caspius*, *Ochlerotatus sticticus*, and *Ochlerotatus dorsalis* [14, 16, 18, 19]. Molecular xenomonitoring can be especially valuable in regions where collecting host infection data is challenging or unavailable [1, 20].

However, the incorporation of community science (or citizen science), a collaborative approach involving the active participation of dog owners and animal shelter volunteers, offers a promising strategy for enhancing *Dirofilaria* spp. infection monitoring. Community science has been proven to be a useful tool in disease ecology and public health and has the potential to overcome limitations such as geographical coverage, resource constraints, and the need for continuous monitoring [21–25]. In the context of dirofilariosis, the contributions of dog owners may provide valuable data on the prevalence, distribution, and risk factors associated with this parasitic infection.

Our goal was to explore the potential of community science for dirofilariosis monitoring in dogs in support of the xenomonitoring approach. Accordingly, by using a questionnaire survey, we collected infection data reported by dog owners, which they previously received from their veterinarians. From these data, we reconstructed country-level distribution patterns, and identified the most important ecological factors (such as age, weight, and owner practices, including the dogs’ lifestyles, among others) that can affect *Dirofilaria* spp. prevalence. Furthermore, we incorporated mosquito xenomonitoring data to get a better understanding of the distribution of dirofilariosis in Hungary. Additionally, we aimed to compare different infection data sets (i.e., community science and xenomonitoring) to test the reliability of volunteer-provided data in dirofilariosis monitoring.

## Methods

### Data collection via community science

A questionnaire was launched in December 2021 and remained accessible until December 2022 on the national mosquito surveillance website (www.mosquitosurveillance.hu). To raise awareness among dog owners, we used various national news and social media platforms. The questionnaire comprised 12 questions, covering topics such as living conditions of the dog, locality, and dog characteristics, including breed, sex, weight, and age. The data were manually reviewed to ensure validity, with questionable responses excluded (e.g., cases where dogs had not been tested for dirofilariosis or that were reported from outside the country).

### Mosquito collection and molecular surveillance of nematodes

BG Sentinel traps (with CO_2_ lure) were placed from 2022 to 2023 at several localities throughout Hungary to collect adult mosquitoes. A total of 138 traps were deployed across 81 cities and towns, with 1–5 traps placed per site depending on local conditions. Mosquitoes were typically collected every 24–48 h, with most traps operating for 2–5 days, although some sites employed traps that remained active throughout the entire season. Afterward, species identification was performed on the basis of available keys [26, 27], and stored in pools of up to 20 individuals at −20 °C until further processing. Samples were extracted using Qiagen Blood and Tissue kit (Qiagen, Hilden, Germany) using the manufacturer’s protocol. For the polymerase chain reaction (PCR), we targeted a 670 bp fragment of the mitochondrial cytochrome c oxidase 1 (*COI*) using general nematode primers. The primers and protocol were used on the basis of previously published work (Casiraghi et al. 2001), using the following primers: COIintF: 5′-TGATTGGTGGTTTTGGTAA-3′ and COIintR: 5′-ATAAGTACGAGTATCAATATC-3′. PCR products were visualized on a 1.5% agarose gel. Positive samples were sent for sequencing to Eurofins Genomics (Koln, Germany).

### Data analysis and visualization

National Center for Biotechnology Information (NCBI) Basic Local Alignment Search Tool (BLAST) was used for species identification of the acquired sequences, after quality assessment. Minimum infection rate (MIR) was calculated to assess infection rate in mosquitoes [28]. Statistical analysis of community science infection data was conducted using a generalized linear model (GLM). A logistic regression model was fit to assess the relationship between the predictors and the likelihood of infection. We used the binary infection status (infected versus noninfected) as the response variable, and the dog’s weight, age, and lifestyle as predictor variables. Weight (in kg: < 5, 5–15, 15–30, 30–50, 50+) and age (in years: < 1, 1–5, 5–10, 10+) were categorized into predefined groups, and dog’s lifestyle was classified as ‘outdoor’, ‘indoor’, or ‘mixed’ when dogs were kept both indoors and outdoors. Data analysis and visualization was performed using R version 4.3.1 [29], using the packages car [30], dplyr [31], emmeans [32], and ggplot2 [33].

## Results

### Distribution of *Dirofilaria* spp. infection using community science data

**Fig. 1:**
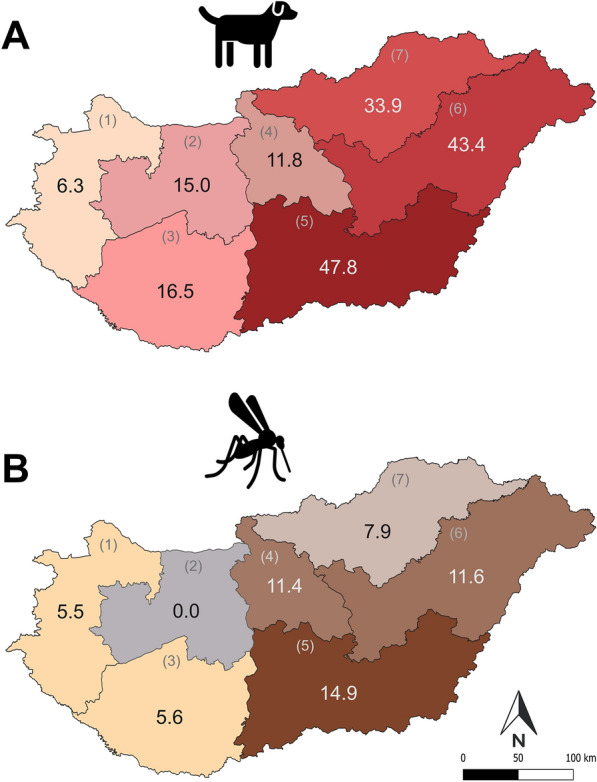
**A, B** Prevalence of dirofilariosis in dogs based on community science data (**A**), and minimum infection rate (MIR) of *D. immitis* in mosquitoes using molecular surveillance (**B**) across seven geographical regions in Hungary. Regions: (1) Western Transdanubia, (2) Central Transdanubia, (3) Southern Transdanubia, (4) Central Hungary, (5) Southern Great Plain, (6) Northern Great Plain, and (7) Northern Hungary

Data for 1661 individual dogs were received from community science participants. After excluding doubtful and incomplete data, 1491 entries remained in the data set, of which 321 showed previous or current infection with dirofilariosis, representing a 21.5% prevalence. Regions in Eastern Hungary showed the highest prevalence rates exceeding 47.8% (Fig. 1A; Table 1). Most cases of infection were reported from Central Hungary (*n* = 87), corresponding to a prevalence of 11.8% (Table 1).

**Table 1:** *Dirofilaria immitis* infection patterns in tested mosquito species and in dogs reported across different geographical regions in Hungary

Regions	*Aedes albopictus*: tested pools/individuals/infected pools	*Aedes japonicus*: tested pools/individuals/infected pools	*Aedes koreicus*: tested pools/individuals/infected pools	*Aedes vexans*: tested pools/individuals/infected pools	Mosquitoes total: tested pools/individuals/infected pools MIR	Dogs: tested/infected Prevalence
1—Western Transdanubia	1/1/0	1/1/0	0/0/0	62/548/3	64/550/3 5.5	80/5 6.3
2—Central Transdanubia	4/5/0	5/5/0	10/19/0	18/41/0	37/70/0 0	133/20 15
3—Southern Transdanubia	50/110/1	3/3/0	19/29/1	21/214/0	93/356/2 5.6	79/13 16.5
4—Central Hungary	144/1441/10	1/1/0	57/166/4	49/238/7	251/1846/21 11.4	736/87 11.8
5—Southern Great Plain	8/69/0	0/0/0	6/12/1	16/53/1	30/134/2 14.9	161/77 47.8
6—Northern Great Plain	3/3/0	0/0/0	12/12/0	20/71/1	35/86/1 11.6	175/76 43.4
7—Northern Hungary	1/1/0	5/6/0	7/8/1	18/112/0	31/127/1 7.9	109/37 33.9

**Fig. 2:**
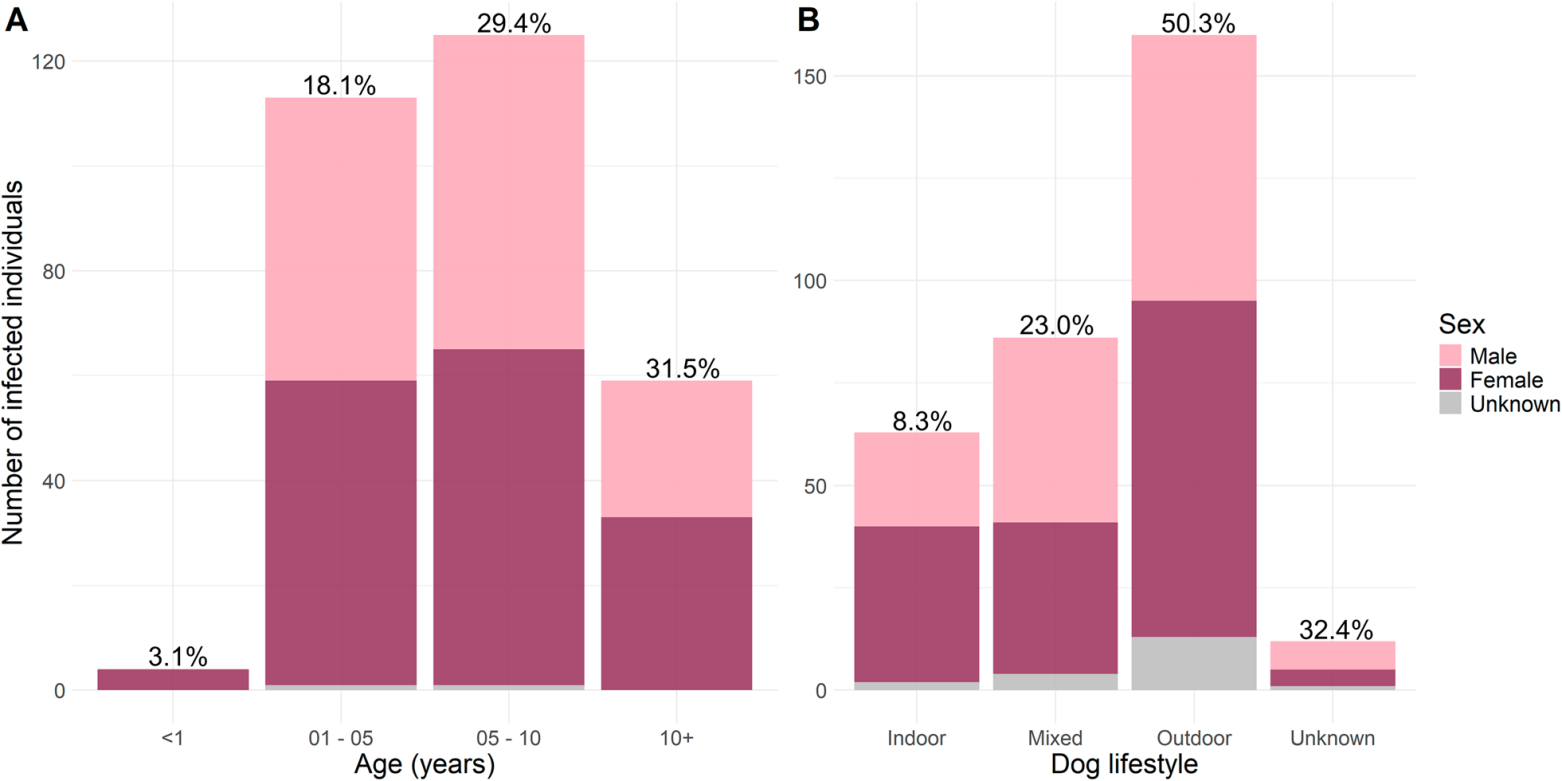
**A, B** Distribution of infection among age groups (**A**) and lifestyles (**B**) of dogs reported through citizen science surveillance. Prevalence of infection is indicated as a percentage above each bar. Indoor indicates dogs kept mostly indoors; mixed indicates dogs kept for an equal time indoors and outdoors; outdoor indicates dogs kept mostly outdoors; and unknown that no data were reported

Infection prevalence was 20.3% and 23.0% in females (*n* = 140/688) and in males (*n* = 161/700), respectively. We found 19.4% prevalence in dogs of unreported sex (*n* = 20/103). Weight did not affect the occurrence of infection. We found a higher likelihood of infection in dogs between the ages of 5 and 10 years (*P* = 0.01; 95% confidence interval [CI] 0.496; prevalence = 29.4%), and older than 10 years (*P* = 0.001; 95% CI 0.757; prevalence = 31.5%), when compared with dogs aged 1–5 years (prevalence = 18.1%). Furthermore, our data showed a significantly lower level of infection in dogs kept exclusively indoors (*P* < 0.001; 95% CI −2.2929; prevalence = 8.3%) or both indoors and outdoors (mixed) (*P* < 0.001; 95% CI −1.1846; prevalence = 23.0%), compared with dogs kept exclusively outdoors (prevalence = 50.3%) (Fig. 2A,B).

### Occurrence of infection in mosquitoes

A total of 541 mosquito pools representing 3169 specimens were tested molecularly; they belonged to four species, including *Aedes vexans* (*n* = 204 pools, *n* = 1277 specimens), *Ae. albopictus* (*n* = 211 pools, *n* = 1630 specimens), *Ae. japonicus* (*n* = 15 pools, *n* = 16 specimens), and *Ae. koreicus* (*n* = 111 pools, *n* = 246 specimens). *Dirofilaria immitis* infection was found in 30 pools of *Ae. albopictus* (*n* = 11 pools, *n* = 102 specimens), *Ae. koreicus* (*n* = 7 pools, *n* = 15 specimens), and *Ae. vexans* (*n* = 12 pools, *n* = 19 specimens) (Table 1). Minimum infection rate by *D. immitis* was 9.4, 6.7, 0.0, and 28.5, for *Ae. vexans*, *Ae. albopictus*, *Ae. japonicus*, and *Ae. koreicus*, respectively.

The highest regional MIRs of *D. immitis* were 14.9, and 11.6 observed in the eastern regions of Hungary (Fig. 1B). Most infections were detected in Central Hungary, including in the capital, with an MIR value of 11.4. Low MIRs or absence of infection was detected in the western regions of Hungary. Overall, *D. immitis* was present in 8 out of 19 counties and the capital region, as well as 6 out of the 7 main geographical regions (Fig. 1B).

Additionally, we detected *D. repens* infection in *Ae. albopictus* (*n* = 1 pool, *n* = 1 specimen; MIR: 0.6), and *Ae. koreicus* (*n* = 1 pool, *n* = 1 specimen, MIR: 4.1). Another parasitic nematode species, *Setaria tundra* was also present in *Ae. vexans* (*n* = 6 pools, *n* = 36 specimens; MIR: 4.7).

### Comparison of xenomonitoring and community science data

Infection prevalence data from community science and MIR from xenomonitoring showed similar geographical distribution patterns, with the highest infection rates observed in the same administrative regions of eastern Hungary (Fig. 1A, B; Table 1). However, xenomonitoring did not detect any infections in Central Transdanubia, despite recent positive reports from community science.

## Discussion

Combining the advantages of community science data and xenomonitoring could increase cost-effectiveness, improve data accuracy, and potentially indicate the spread of emerging pathogens. Here, we show that it is possible to integrate xenomonitoring with community science data when the aim is to investigate the geographical distribution patterns of the disease. Community science data could not only improve our understanding of pathogen occurrence but also shed light on infection patterns in hosts. For instance, dogs kept outdoors exhibit higher levels of *Dirofilaria* spp. infection compared with those primarily kept indoors. This may result from greater exposure to vectors for dogs kept outdoors, placing them at a higher risk of infection, which has been observed in previous studies, as well [34–36]. Age also seemed to be a contributing factor to the presence of infection, as older individuals were more likely to be infected, likely due to prolonged exposure to both the mosquito vector and the parasites, as well as the long prepatent period of the infection [12, 37–39]. We found that weight had no effect on infection patterns.

Invasive mosquitoes have been suggested to play a role in *Dirofilaria* spp. transmission, which has been proven both under natural and laboratory conditions [40–42]. Additionally, invasive mosquitoes are occasionally found to be infected by *Dirofilaria* spp. during pathogen surveillance and xenomonitoring [19, 43]. Here, we found the highest MIR in the invasive *Ae. koreicus*, further indicating its role as a vector. Furthermore, we also found the presence of *D. immitis* in the native species, *Ae. vexans* and the invasive *Ae. albopictus*, highlighting that both native and invasive species may contribute to *Dirofilaria* spp. circulation in Hungary. We also found the presence of *D. repens* in invasive mosquito samples. The first autochthonous infections in domestic dogs caused by *D. repens* were reported in 1998 in Hungary [44]. A previous study has shown that the prevalence of *D. repens* in dogs was 14.2% in 2017 [5]. This infection is considered an emerging zoonosis in Europe and is occasionally associated with human infections in Hungary, as well [45]. Our results confirm previously observed patterns in Central Europe, where *Ae. vexans* and *Ae. koreicus* are commonly found to be infected during xenomonitoring of *Dirofilaria* parasites [14, 18, 19, 46–48].

We found the presence of the emerging nematode species, *S. tundra* in *Ae. vexans*, which is the causative agent of setariasis in various cervid species [49]. This finding suggests its potential vectorial role, as *Ae. vexans* has previously been shown to exhibit a high infection rate with this nematode species within the country [14], and is showing an emerging infection rate across Europe [50–52].

The higher infection rates in both mosquitoes and dogs in the eastern and southern parts of the country can be attributed to a combination of factors. In these regions, we received proportionally more reports of dogs being kept outdoors (Supplementary Materials Table S1), indicating that this owner habit is more prevalent compared with other areas, which likely increases dogs’ exposure to infected mosquitoes. Nevertheless, there is limited information about preventative measures, such as parasite control in these regions. Furthermore, the infection rate in local wildlife, which can act as a reservoir for *Dirofilaria* species, might also be high in these regions [9], which can contribute to additional interspecies pathogen flow between wildlife and domestic dogs [53, 54]. Environmental conditions such as warmer temperatures, which can create ideal conditions for both mosquito breeding and parasite development, likely contribute to higher infection rates, as well [5]. Moreover, the composition and abundance of vector species may vary between regions, potentially favoring those with greater vectorial capacity [55]; however, available data on this are limited. Additionally, cross-border transmission is likely playing a role in the spread of *D. immitis*. In Romania, counties near the southern Hungarian border, such as Timis, show the highest rates of *Dirofilaria immitis* infection in dogs [56]. Similarly, northern regions of Serbia, particularly around areas with high infection rates in Hungary, also report elevated infection levels. In Kikinda, Serbia, the highest proportion of dogs were found to be microfilaremic, with *D. immitis* being the most prevalent parasite in the region, present in 16.1% of dogs, compared with other screened regions in Serbia [57]. Although travel history may also contribute to cross-border infections, we did not collect information on the dogs’ movements, as *Dirofilaria* infection is considered endemic in Hungary; nonetheless, this may represent a limitation of the study. Future research should investigate the role of cross-border transmission in the spread of infection. Overall, dog ownership practices, the local environment, the climate, and both interspecies and intraspecies parasite transmission may all contribute to an increased overall risk of *Dirofilaria* infection in mosquitoes and dogs in these regions.

Effective tracking of disease dynamics in both hosts and vectors requires the use of combined methods that are reliable, cost-effective, and widely applicable, such as community science and xenomonitoring. Understanding infection spread and disease patterns is necessary to improve preventative efforts and control methods. Involving local communities in data collection can improve awareness of zoonotic parasitic diseases such as dirofilariosis, emphasizing their importance and leading to a better understanding of their spread. This increased awareness among dog owners could, in turn, support more effective prevention and management strategies, given that controlling dirofilariosis largely depends on their actions [58, 59]. It is important to acknowledge that community science data may include false-positive results for *D. immitis*, as some laboratory tests, such as antigen or antibody tests, Knott’s test, or blood smear, occasionally cannot distinguish between *D. immitis* and *D. repens* (or other microfilariae) due to potential crossreactivity. Furthermore, *D. immitis* is likely more frequently diagnosed by veterinary clinicians because of its link to prominent cardiovascular symptoms, while *D. repens* may be overlooked due to the lack of such noticeable clinical signs. As a result, the distribution of *D. immitis* could be overestimated, whereas the occurrence of *D. repens* could be underreported.

## Conclusions

Xenomonitoring and community science have proven highly effective in strengthening disease surveillance efforts in domestic animals. Both methods offer complementary but distinct types of disease ecology data, each with its own limitations, with xenomonitoring providing time- and location-specific insights, while community science offers broader, retrospective information that may be less suited for assessing current epidemiological trends. Targeting volunteer data providers, here dog owners, could greatly improve our understanding about infection patterns and disease ecology in domestic dogs. In the context of large-scale epidemics or widespread outbreaks, it can play an important role in informing and supporting preventive measures. While veterinarians may have access to some of these data, much of the information, particularly about dog ownership habits, is typically not documented. By developing partnerships between the scientific community and dog owners, we can increase the collective power of community science to advance our knowledge of *Dirofilaria* spp. eco-epidemiology, ultimately improving the health and well-being of both pets and humans in affected regions. Xenomonitoring is an effective tool for developing early defense strategies, as it can detect pathogens in vectors before they lead to epidemics in hosts such as domestic dogs, particularly in the case of emerging pathogens originating from wildlife. In contrast, citizen science typically identifies infections after they have occurred, making it less suitable for early prevention at the individual level. Combining these different methods has proven useful not only for tracking the current spread of infection but also for assessing the risk of larger-scale epidemics, such as those associated with the movement of pets to other regions. Moreover, both approaches provide valuable insights into the quality and effectiveness of existing disease control and prevention measures in both vectors and hosts, highlighting areas where further implementation may be necessary. Overall, our findings highlight that an integrated approach to national disease monitoring serves as a valuable alternative when direct pathogen screening methods are limited or unavailable.

## Supplementary Information

Supplementary Material 1.

Supplementary Material 2.

## Data Availability

Data are provided within the manuscript or supplementary information files.
